# Two-stage cluster sampling to assess SARS-CoV-2 seroprevalence without pre-enumeration: An example from Madagascar

**DOI:** 10.1371/journal.pone.0334627

**Published:** 2025-11-04

**Authors:** Eva Lorenz, John Amuasi, Tiana Randrianarisoa, Tahinamandranto Rasamoelina, Leonard Gunga, Dominik Benke, Jonathan Ströbele, Jenny Kettenbeil, Wibke Loag, Haja Andriamahandry, Landry Razanakolona, Jean Rolland Randrianirina, Hosea Randrianasolo, Jean Christian Ratombotsoa, Fitahina Nahita, Daniel Eibach, Daniela Fusco, Jürgen May, Rivo A. Rakotoarivelo, Aurélia Souares, Emmanuel Bonnet, Nicole S. Struck

**Affiliations:** 1 Infectious Disease Epidemiology, Bernhard Nocht Insitute for Tropical Medicine, Hamburg, Germany; 2 German Center for Infection Research (DZIF), Hamburg-Borstel-Lübeck-Riems, Heidelberg, Germany; 3 Research Group Global and One Health, Bernhard Nocht Insitute for Tropical Medicine, Hamburg, Germany; 4 Kumasi Centre for Collaborative Research in Tropical Medicine, Kwame Nkrumah University of Science and Technology, Kumasi, Ghana; 5 Department of Global and International Health, Kwame Nkrumah University of Science and Technology, Kumasi, Ghana; 6 University of Fianarantsoa, Fianarantsoa, Madagascar; 7 Centre d’Infectiologie Charles Méreiux, Antananarivo, Madagascar; 8 Department of Tropical Medicine I, University Medical Center Hamburg-Eppendorf (UKE), Germany; 9 Heidelberg Institute of Global Health (HIGH), Heidelberg University Hospital, Heidelberg University, Heidelberg, Germany; 10 Institut de recherche pour le développement, IRD, UMR 215 Prodig, 5, cours des Humanités, Aubervilliers, France; Institut Pasteur de Madagascar, MADAGASCAR

## Abstract

Implementing population-based surveys in resource-constrained settings presents logistical challenges when detailed population enumeration is unavailable. We developed a field mapping system integrated into a cluster sampling approach to eliminate pre-enumeration requirements for a SARS-CoV-2 seroprevalence survey in Madagascar. We conducted a cross-sectional observational study in urban Fianarantsoa, Madagascar, between February and June 2021. Using probability proportional to size sampling, we selected clusters from administrative areas (fokontany) and randomly generated GPS coordinates within these clusters. Field teams navigated to coordinates using OpenStreetMap software on tablets, identified eligible households, and conducted health surveys with blood sampling. We employed a mobile-compatible system for real-time household mapping and data collection, functioning without continuous network connectivity. Sample size calculation targeted 650 households (SARS-CoV-2 seroprevalence 30%, precision ±5%, design effect 2.0). Our specific objectives were to develop and implement a geographic cluster sampling method that did not require pre-enumeration; to assess the feasibility of this method through participation rates; and to evaluate potential selection biases related to socioeconomic factors. We identified households at 95.3% (696/730) of randomly generated GPS coordinates. Of contacted households, 96.8% (674/696) participated, representing 1,121 individuals across 57 clusters. Participation rates varied geographically, with a modest inverse correlation with household wealth (participation decreased by 0.85% per wealth quintile increase, 95% CI: −3.54% to 1.84%). Demographic characteristics of our sample matched census data for urban Fianarantsoa, supporting the representativeness of our approach. This integrated field mapping system created a virtual household map simultaneously with survey implementation, enabling cost-effective two-stage cluster sampling without pre-enumeration. The approach enabled evaluation of selection bias, simplified logistics, and provided a permanent geo-referenced database of surveyed households. This methodology offers a practical solution for population-based surveys in resource-constrained settings with incomplete enumeration data and has applications beyond COVID-19 research for various public health surveillance activities.

## Introduction

Estimates of severe acute respiratory syndrome coronavirus type 2 (SARS-CoV-2) seroprevalence are important for decision-makers at global, national and subnational levels to prioritise public health interventions, vaccine introduction and resource allocation. However, accurate population-level prevalence estimates are scarce, especially in resource-limited settings [1]. The SeroCoV project is a multi-country study (Burkina Faso, Ghana and Madagascar) that aimed to help close this gap [2]. Common approaches to assessing SARS-CoV-2 seroprevalence include facility-based and population-based studies. While facility-based studies are more commonly conducted due to their operational simplicity, they have inherent limitations in population representativeness as they primarily capture patients actively seeking care at study facilities, potentially overrepresenting those with severe symptoms and healthcare access [3]. In contrast, population-based studies provide direct measurements of disease prevalence in the general population, but remain uncommon due to the substantial resources requirements and complex field implementation demands [4]. They can be further complicated in areas with rapidly changing or hard-to-reach populations or incomplete enumeration coverage of the underlying population [5–7]. A systematic review by Bobrovitz et al. [8] found that only 22% of SARS-CoV-2 seroprevalence studies worldwide used population-based probability sampling approaches, while the majority relied on selective convenience samples from blood donors, healthcare workers, or outpatients.

While geospatial approaches to survey sampling have been documented in previous studies, including GPS-based sampling and gridded population methods [9–11], methodological gaps remain for rapid implementation during health emergencies. Traditional approaches often require extensive pre-enumeration or high-resolution satellite images, limiting their feasibility when time constraints are critical. Recent advances in geospatial sampling methods have demonstrated the feasibility of selecting households via satellite in various settings, including rural areas of Guatemala and Mozambique [12,13]. The SeroCoV project employed an identical two-stage cluster sampling methodology at all study sites in Burkina Faso, Ghana and Madagascar. This article presents Madagascar as a detailed methodological case study because the country exemplified the full spectrum of operational challenges that this approach addresses, including mountainous terrain, limited infrastructure and data, and the need for rapid pandemic implementation. This single-site analysis allows for comprehensive operational documentation.

We describe the geographic cluster sampling method used in Madagascar for the SeroCoV survey with the aim of enhancing population representativeness of estimates and to share our experiences and lessons-learnt to inform future research. The SeroCoV site in Fianarantsoa, Madagascar, included areas with low population density and difficult to traverse mountainous terrain which made a preliminary survey to identify and map households time-consuming and costly. We therefore developed a geospatial survey to map and manage the survey in real time. As the research assistants were able to map in the field during the survey, the system eliminated the need for a pre-enumeration and allowed for a continuous survey throughout the study period.

The specific objectives of this study were to: (1) develop and implement a cost-effective geographic cluster sampling method without requiring pre-enumeration; (2) assess the feasibility and acceptability of this approach through household participation rates and field team experiences; and (3) evaluate potential selection biases in this sampling method, particularly related to socioeconomic factors.

## Methods

### Study site

This study was conducted in the urban residential area (i.e., the study site) of Fianarantsoa in Madagascar in the context of the multi-country SeroCoV study [2]. The study site was selected in consultation with local investigators based on laboratory infrastructure, community accessibility and staff safety. An urban site was preferred over a rural site because more cases were registered in the official systems in urban areas. Fianarantsoa is the capital of the Haute Matsiatra region in south-central Madagascar at an average altitude of 1,200 metres with a population of approximately 190,000 inhabitants. The age distribution is typical for countries in sub-Saharan Africa, with a median age of 19.0 years, and a Gross National Income per capita of USD 490 in 2021 [14–16].

### Design

Details of the general SeroCoV methods, including the household survey and laboratory analyses, are described elsewhere [17]. In brief, a two-stage geographical cluster sampling procedure was used to identify eligible households for participation in the survey, which included both household mapping and participant selection. Participation was voluntary, and at the end of a household visit, participants were offered a compensation of one kilogram of rice and sugar per household, or rice only in the case of an existing contraindication. Household members were included after informed consent was obtained. The minimum age was 10 years and there had to be no health problems that prevent blood sampling. Household members who met these criteria were presented with a structured questionnaire to assess related factors, such as sociodemographic information, previous episodes of illness, and history of testing and treatment for COVID-like symptoms (Questionnaire in Supporting Information S2 File).

### Ethics approval and consent to participate

All experimental protocols were approved by the Department for Infectious Disease Epidemiology, Bernhard Nocht Institute for Tropical Medicine, Germany. The SeroCoV study protocol, questionnaire, information leaflet, and informed consent forms were reviewed and ethical clearance was obtained by the National Ethical Committee, Ouagadougou, Burkina Faso (Reference No: 2020 137/MS/SG/INSP/CRSN); Ministry of Public Health in Antananarivo, Madagascar (Reference No: CERBMIORG0000851, No 175-MSANP/SG/AGMED/CNPV/CERBM); Committee on Human Research, Publication and Ethics in Ghana (Reference No: CHRPE/AP/218/20), and the Ethical Commission of the Ärztekammer Hamburg, Germany (2020–10035-BO and 2020–10035–1-BO). Informed consent to participate was obtained from all of the participants in the study. In case individuals were younger than the age of 18, consent to participate was obtained from their parents or legal guardians.

### Cluster randomization

**Fig 1:**
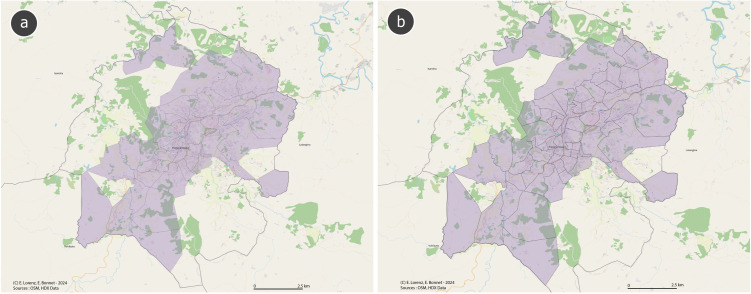
A map of (a) fokontany in Fianarantsoa and (b) a differentiation of included clusters within fokontany. Map created with Quantum Geographic Information System (QGIS) software using OpenStreetMap data (OpenStreetMap contributors, ODbL license) and administrative boundaries from OCHA HDX (CC BY-IGO license).

Background information on population size and administrative boundaries was available through subnational population statistics provided by the UN Office for the Coordination of Humanitarian Affairs and used for the weighted sampling strategy [18,19]. Fianarantsoa district includes 50 fokontany, of which 17 fokontany were excluded on the advice of the local team prior to sampling due to poor accessibility in remote areas with limited road access. According to available census data, these areas had a combined population of around 20% of the district’s total population. The so-called fokontany are the smallest administrative unit in Madagascar and consist of one or more villages. A map of the catchment area was overlaid with a virtual layer containing the administrative boundaries of the 33 fokontany in question (Fig 1a). We chose the administrative boundaries to account for different population sizes, as it is important that every household in each of these areas had an equal chance of being selected for the study, regardless of population density. Administrative areas were sampled using the probability proportional to population size (PPS) method whereby the selection probability increased with the population size of the administrative area [20,21]. The population sizes per eligible fokontany ranged from 1,171–15,588 inhabitants resulting in an allocation of 1–4 clusters per fokontany using a custom routine implemented in R, version 3.5.1, consistent with the PPS. The administrative areas per fokontany were segmented into the assigned number of clusters with approximately equal average population using a Voronoi algorithm (Fig 1b) [22]. The algorithm was run on the centroids determined by k-means clustering of uniformly distributed random coordinates within each administrative area. In determining the segment size, a balance was struck between the desire to reduce cluster effects and efficiency related to field team travel times and low household density. The mean cluster area (standard deviation, range) was 0.65 (0.72, 0.07–3.41) km^2^. A sample size estimation was performed prior to the study. Assuming a SARS-CoV-2 seroprevalence of 50%, a precision of ±5%, a design effect of 1.45 to account for cluster sampling, and a 15% non-response rate, 655 households were required.

**Fig 2:**
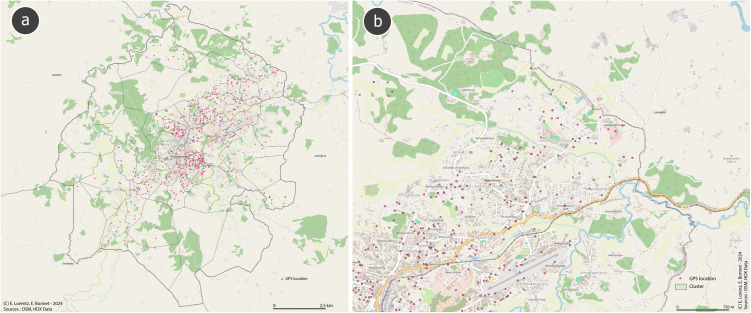
Fianarantsoa with (a) catchment fokontany with randomly allocated GPS coordinates including 30% backup and (b) example segmentation of one Fokontany with underlying road infrastructure. Map created with Quantum Geographic Information System (QGIS) software using OpenStreetMap data (OpenStreetMap contributors, ODbL license) and OCHA administrative data (CC BY-IGO license).

After the clusters were created, a list of random global positioning system (GPS) coordinates for potentially eligible household locations was generated using Quantum Geographic Information System (QGIS) software in the second stage of sampling. A further 30% of the GPS coordinates were created as backup in case no household could be identified at the randomly assigned location or if households refused to participate (Figs 2a and 2b).

### Field surveying

**Fig 3:**
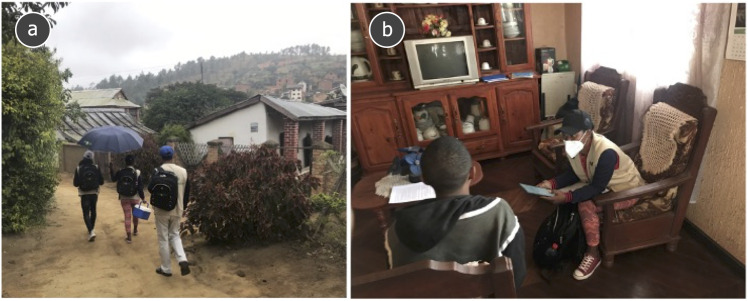
(a) SeroCoV field team (study nurses and physician with SeroCoV umbrella and sample cooling box) in the process of navigating to a household coordinate in May 2021. (b) Tablet-based survey conduct and data entry including household mapping.

In Fianarantsoa, the field team used tablet-based OpenStreetMap Automated Navigation Directions (OsmAnd) software to navigate to the coordinates and identify eligible households according to standardised protocols [23]. The tablets used a GPS sensor for navigation. After arriving at a GPS coordinate, the teams identified the households in question according to predefined standard operating procedures (SOP), taking into account different scenarios: A GPS coordinate falling on exactly one household, multiple households, or no household (SOP for field navigation in Supporting Information S1 File). Households were defined as groups of people living together and sharing meals (excluding residential facilities such as boarding schools, dormitories, hostels, prisons and other communities hosting grouped people). All households that had a front door and a consenting adult aged ≥18 years were eligible for participation. If the household residents were not present at the time of the visit, the local team returned either on the same day while still in the area or at a later date (maximum two weeks) to attempt recruitment. The on-site surveys were carried out by two local teams. Each team consisted of a doctor, two nurses and a driver (Figs 3a and 3b).

The medical staff were native Malagasy speakers and fluent in French. Field teams underwent a comprehensive training program conducted by the principal investigator and study coordinator. The training covered good clinical practice, study protocol implementation, proper household selection procedures, ethical consent processes, standardized questionnaire administration, blood sample collection and handling techniques, and electronic data capture using the REDCap® Mobile App (Research Electronic Data Capture) [24].

A paper-based list was used to ensure traceability of participating households and to match the blood samples with the survey and household demographics. The list contained the date, name and address of the participant, the Screening Identifier, the Household Identifier, the Member Identifier and checkboxes, indicating whether informed consent, blood sampling and questionnaire had been completed. In addition, any comments were noted in an open text section. A supervisor prepared weekly survey schedules for the local teams and monitored progress using Microsoft Excel. For quality assurance, a clinical research assistant verified data entries on a daily basis. Weekly reports with stratification targets (population stratified by age and sex) were generated to monitor the completeness of the surveys in the clusters. The reports were shared with both local coordinator and the principal investigator and discussed in biweekly online team meetings. The data was regularly checked for completeness and the total number of households surveyed was routinely verified against household survey data collected. This system allowed supervisors to monitor progress and make adjustments as needed. For example, there were some initial difficulties in recruiting men due to their absence during the day, which led to a change in the survey schedule to take account of their availability. As the local team drove and walked through the clusters, they marked each enrolled household using geo-referenced pins in REDCap. The database also allowed to indicate their participation status and included a series of questions and notes to facilitate return visits if necessary (e.g., contact number and best time of day for a return visit). Data was synchronised daily with the REDCap server each afternoon as the local teams returned and the tablets were connected to wireless internet.

To minimize selection bias, teams were instructed to make up to three contact attempts at different times of day and different days of the week (including evenings and weekends) before classifying a household as non-responsive. This approach was particularly important for ensuring adequate representation of working adults who might be absent during standard working hours. Between February and June 2021, two field teams conducted the survey over a period of 16 weeks. Teams operated five days per week, completing an average of 8–10 household surveys per team per day (combined weekly average of 40–50 households).

### Statistical analyses

To assess the response rate and representativeness, the response rate in the survey was calculated by dividing the number of successfully surveyed households by the total number of households identified and approached per cluster. We then aimed to examine possible biases in household participation and assumed that wealthier households were less likely to participate in the study. To estimate household wealth, we derived a household wealth index using principal component analysis of assets of households participating in the survey, including sanitary facilities, electricity, or ownership of electronic devices. However, we lacked data on households that declined to participate in the study. We used linear regression to assess the association between survey participation rate and average household wealth, both at the cluster level. PPS, descriptive statistics, map creation, and regression were performed using R software, version 4.0 and 4.3.1 or higher and QGIS software, version 3.14 or higher. Segmentation of fokontany was done using Python, version 3.11.7 or higher.

## Results

**Fig 4:**
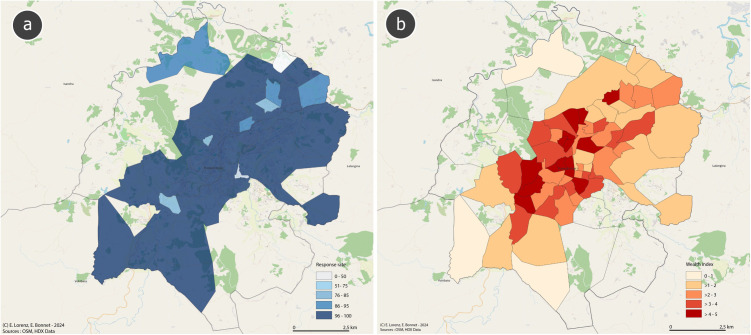
Map of Fianarantsoa I sampling areas showing (a) survey response rate and (b) average household wealth index (quintile, with 5 the wealthiest). Map created with Quantum Geographic Information System (QGIS) software using OpenStreetMap data (OpenStreetMap contributors, ODbL license) and OCHA administrative boundaries (CC BY-IGO license). Data in Supporting Information S3 File.

The local teams navigated to 730 GPS coordinates, of which 34 did not allow household identification either because no household was within 100 meters, a building was identified but no respondent. Overall, the rate of identifying households at GPS coordinates was 95.3% (696 of 730). The remaining 696 households were contacted and 674 agreed to take part in the study. The proportion of households declining participation was 3.2% (22 of 696) with the majority of cluster having response rates above 96% (Fig 4a). The enrolled households contained 1,121 individuals. Households from 57 clusters were enrolled with 12−13 households per cluster. Areas with higher average wealth quintiles very weakly correlated with lower participation rates. Participants with higher household wealth tended to live the centre of Fianarantsoa, while participants from less wealthy households tended to live in the surrounding area (Fig 4b). The regression model showed that the participation rate decreased by −0.85% (95% confidence interval, −3.54–1.84) for each quintile increase in wealth.

## Discussion

Conducting population-based household surveys in resource-constrained settings presents logistical challenges, particularly when detailed infrastructure maps are outdated and/or prior household enumeration is not available. We integrated a household survey with biological sampling into a two-stage cluster random sampling scheme to survey 674 households in Fianarantsoa, Madagascar, without pre-enumeration data.

Previous approaches have used satellite-derived building footprints requiring accurate data and single-family occupancy assumptions [25,26], or systematic proximity methods with varying selection requirements [27]. Another sampling technique involved initiating surveys from patients recently diagnosed with enteric fever within the community which can be prone to selection bias [28]. Alternatively, random coordinate selection may oversample low-density areas and introduce bias in mixed-density settings with multiple households per location [29].

Our approach builds on recent methodological advances in geospatial community sampling [4,30] and provides an alternative approach that addresses some of these limitations. Eliminating the pre-enumeration step significantly reduced the time taken to implement the system during the pandemic response. Our integrated approach enabled the real-time creation of georeferenced household databases during the implementation of the survey, in a manner similar to the dynamic mapping concept demonstrated in Nepal [4] which we adapted to the specific challenges of SARS-CoV-2 seroprevalence assessment in Madagascar. The permanent database of sampled households could potentially support future public health initiatives in the same communities. Unlike approaches that require extensive processing of satellite imagery [31,32], our method prioritises immediate implementation capability which enabled immediate quality control and progress monitoring, both of which were essential during the pandemic. Our approach combines cluster selection based on administrative boundaries with real-time field mapping without relying on gridded population data or satellite-derived building footprints [9,11]. This addresses the issue of outdated or incomplete building footprint data while maintaining probability-based sampling principles.

### Strengths

Real-time creation of georeferenced household databases enabled immediate quality control and progress monitoring. It enabled continuous sampling throughout the study period, providing quality control and progress monitoring capabilities that are not possible with paper-based systems. The permanent database could support future public health initiatives in the same communities. Using open-source tools (QGIS and OsmAnd) reduces dependency on proprietary data sources, making the methodology accessible to research projects with limited funding. The practical application in Fianarantsoa was highly efficient, achieving 95.3% successful household identification at GPS coordinates and a participation rate of 96.8%. The demographic characteristics of our sample matched census data, which supports the representativeness of our approach. Standardised household selection procedures reduced the risk of selection bias arising from subjective decisions made by survey teams. This methodology paper complements other SeroCoV publications by providing detailed operational guidance on the sampling approach used at all sites. The main SeroCoV results papers, meanwhile, focus on epidemiological findings and cross-country comparisons. This single-site documentation serves the broader research community by offering a replicable framework for conducting similar surveys in settings with limited resources.

### Limitations

Our geosurvey enabled local teams to create a dynamic, up-to-date, geotagged map of the households targeted locally. However, its use is subject to issues related to survey preparation (requires geographical information on a low administrative level in the form of vector data of regions together with population and demographic data), technological limitations (requires devices that allow navigation and data entry), and on-site household selection (requires standardised household selection procedures, and in less densely populated areas, local teams may survey many GPS coordinates that are not located in populated neighbourhoods). Additionally, applying this method in remote areas poses challenges related to the boundary delineation process, the use and translation of satellite images between GIS and GPS, and household selection at each survey point under different field conditions.

The survey was susceptible to selection bias from informal housing and GPS inaccuracies in densely built areas [33]. We found evidence that households in wealthier areas were less likely to participate in the study. Because care-seeking behaviour and disease risk are often associated with household wealth, quantifying participation biases in these surveys may allow adjustments to produce more accurate and representative estimates of care seeking or disease burden. Our approach may not be directly transferable to rural settings with much lower population densities and fewer recognisable landmarks for navigation. While our participation rate was high, we cannot rule out selection bias, particularly as we could not directly assess characteristics of non-participating households. The use of GPS technology introduces potential inaccuracies, particularly in densely built urban areas where satellite signals may be compromised by high buildings. The exclusion of 17 fokontany due to accessibility constraints represents a significant limitation affecting generalisability to the most remote or underserved populations within the district. Detailed demographic data for these areas was not systematically collected prior to the decision to exclude them which limits our ability to quantify the introduced bias [34]. These limitations reflect broader challenges in population-based survey methodology identified in recent literature such as the critical importance of defining representativeness in study samples, particularly when population enumeration is incomplete [35]. The tension between operational feasibility and statistical representativeness remains a key challenge in global health research [36].

### Future directions

Future research should focus on adapting this methodology to diverse settings, particularly rural areas with limited geographical data and integration with mobile health technologies. Recent advances in machine learning-based population mapping [37] and digital transformation initiatives in public health [38] could enhance real-time survey monitoring systems while supporting local research capacity development [39,40].

## Conclusion

Our integrated field mapping approach successfully addressed the challenge of conducting population-based seroprevalence surveys without pre-enumeration in resource-constrained settings. The method achieved high participation rates (96.8%) and demographic representativeness. While developed for SARS-CoV-2 seroprevalence assessment during the early pandemic response, the methodology has broader applications for public health surveillance activities in similar settings.

## Supporting information

S1 File Standard Operating Procedures for field navigation. Detailed protocols for household identification and selection procedures used by field teams when GPS coordinates corresponded to different scenarios (single household, multiple households, or no household present).
(PDF)

S2 File Household survey questionnaire. Complete questionnaire used to assess sociodemographic information, previous illness episodes, and COVID-19 testing and treatment history among study participants.
(PDF)

S3 File Cluster-level summary statistics of population sizes, participation rates, wealth indices, age and sex distribution. (CSV)

## Abbreviations:

COVID-19: Coronavirus disease 2019
GPS: Global Positioning System
OsmAnd: OpenStreetMap Automated Navigation Directions
PPS: Probability Proportional to population Size
QGIS: Quantum Geographic Information System
REDCap: Research Electronic Data Capture
SARS-CoV-2: Severe Acute Respiratory Syndrome CoronaVirus type 2
SOP: Standard Operating Procedures
TSAP: Typhoid Fever Surveillance in Africa Program
