# Supplementary material for: Two-stage cluster sampling to assess SARS-CoV-2 seroprevalence without pre-enumeration: An example from Madagascar
Source: PLoS One. 2025 Nov 4;20(11):e0334627. doi: 10.1371/journal.pone.0334627 (PMC12585014; doi:10.1371/journal.pone.0334627)
Supplement: S1 File — Detailed protocols for household identification and selection procedures used by field teams when GPS coordinates corresponded to different scenarios (single household, multiple households, or no household present). (PDF) [file pone.0334627.s001.pdf]

**Seroprevalence of SARS-CoV-2 in three African Countries (SeroCoV)**<br>Burkina Faso – Ghana – Madagascar<br>KCCR	SeroCoV
**Title: Standard Operating Procedure (SOP) for Navigation and Household identification**	
**Authors:** Eva Lorenz, Anna Ginsbach	

This SOP starts after you installed OsmAnd on your tablet and set up your site-map with the GPS respective coordinates. Internet access is only necessary to synchronize your entered and saved data with the server in Hamburg.

Step 1: Navigate to GPS coordinates.
To start the navigation, you need to use the navigation button on the map screen. Next, you'll need to set the starting point and the destination. As for the starting point, please choose your current location. To navigate to a first GPS point from the file (choice of the starting GPS coordinate is up to you), just press the navigation button in its context menu. As soon as your point is selected, the app will create a route and will start guiding you after you tap Start. You can already add select several of the points to plan ahead the route for the next couple of days. To navigate to the second and subsequent GPS point from the .gpx file (choice of the starting GPS coordinate is up to you), just press the navigation button in its context menu. As soon as your points are selected, the app will create a route as before and will start guiding you after you tap Start.

Step 2: Save real GPS location of identified households.
Once you reached a GPS coordinate from the list and managed to identify a respective household (see instructions in Table 1): Save your current location, click on 'update GPS data' in REDcap to update the GPS coordinates.

To synchronize the entered and locally saved data with the server in Hamburg, an Internet connection is again needed (see SOP 'Data synchronization').

After successful navigation to a GPS coordinate based on the previous instructions, the following steps are needed to identify a household for study participation:

**Scenario 1:** GPS coordinate falls on exactly one household	**Scenario 2:** GPS coordinate does not fall onto household	**Scenario 3:** GPS coordinate falls on a building with multiple households
Save the location in REDCap (upload 'real GPS') in database record with respective screening ID.	Identify closest household within a radius of 100 meters.	Go to the ground floor. Select household with first door on your right. Turn clockwise until you find door. If no identifiable household move the next floor and repeat procedure until the top floor is reached.
If no one is at home, it may be possible to ask neighbours whether any eligible respondents live in the household. If yes, come back to this household at a later point; either on the same day while you are still in the area, or at a later time point (maximum two weeks).	If no household is identifiable within a radius of 100 meters, navigate to the next GPS coordinate on your map and continue again with scenarios 1, 2 or 3.	If no household is identifiable, navigate to the next GPS coordinate on your map and continue again with scenarios 1, 2 or 3.
If eligible respondent is at home, please introduce yourself and the study and continue with SSP/SOP interview	If several appear to be similarly close: face north, from there turn 45° clockwise and select the first on your right. Continue with 1 or 3.	If household was successfully identified, ave the location in REDCap (upload 'real GPS') in database record with respective screening ID.
If not, continue to navigate to the next GPS coordinate on your map and continue again with scenarios 1, 2 or 3.		

Table 1. Instructions to identify eligible households.
