# Supplementary material for: Two-stage cluster sampling to assess SARS-CoV-2 seroprevalence without pre-enumeration: An example from Madagascar
Source: PLoS One. 2025 Nov 4;20(11):e0334627. doi: 10.1371/journal.pone.0334627 (PMC12585014; doi:10.1371/journal.pone.0334627)
Supplement: S2 File — Complete questionnaire used to assess sociodemographic information, previous illness episodes, and COVID-19 testing and treatment history among study participants. (PDF) [file pone.0334627.s002.pdf]

	STUDY CENTRE:	SeroCoV Study Forms<br>Case Report Form (Household)	|_||_|/|_||_|/|_||_| (DD/MM/YY)

H_01	**Screening ID:**
H_02	**Household ID:**
H_03	**Recruitment Date:** |___|___|/|___|___|/|___|___| (DD/MM/YY)
H_04	**Data collector initials:** |__||__||__|

Participation

H_05	**Will the household participate?** ☐ Yes ☐ No
H_06	**If no, why not?**<br>☐ No contact ☐ Distrust ☐ Other
H_07	**If other, please specify:**
H_08	**Remarks:**

Household information

H_10	**If Ghana,** ☐ Accra ☐ Kumasi
H_13	**If Kumasi,**<br>☐ Asokwa ☐ Tafo ☐ Bantama ☐ Nhyiaeso ☐ Subin<br>☐ Kwadaso, ☐ Suame ☐ Oforikrom ☐ Asawase ☐ Manhyia<br>☐ Other<br>If Other, please specify: ________________________________
H_15	**Corrected GPS:**<br>North/South: |___|.|___|___|.|___|___||___| °N<br>West/East: |___|.|___|___|.|___|___||___| °W
H_16	**Telephone number:**

Corona baseline

H_19	Did/Do you have a suspected case in your household? *("Suspected case" has to be explained to participant)*<br>☐ Yes ☐ No ☐ Don't know
H_20	How many cases in your household have been confirmed? *("Confirmed case" has to be explained to participant)*<br>☐ One person ☐ More than one ☐ The entire household ☐ Don't know
H_21	Was anyone in your household treated for a febrile illness since the beginning of the Corona-crisis?<br>☐ Yes ☐ No ☐ Don't know
H_22	If yes, what were they treated for?<br>☐ Malaria ☐ Flu ☐ Don't know ☐ Other
H_23	If other, please specify:

Household and living conditions

H_24	House occupation status:<br>☐ Owner ☐ Renting ☐ Accommodation from employer<br>☐ Living at parents / friend's house ☐ Others
H_25	If other, please specify:
H_26	What is the total number of occupied rooms? |___||___|
H_27	What is the average number of people sleeping in one room? |___||___|
H_28	Type of toilets:<br>☐ Water closet (WC) ☐ Latrines with ventilating pit ☐ Ordinary latrine<br>☐ Open defecation ☐ Other
H_29	If other, please specify:
H_30	What is your main energy source for cooking energy?<br>☐ Electricity ☐ Gas ☐ Coal / wood ☐ Oil stove ☐ Other
H_31	If other, please specify:
H_32	What main source of lighting energy do you use?<br>☐ Electricity ☐ Gas ☐ Storm lamp (Lantern) ☐ Solar lamp ☐ Other
H_33	If other, please specify:
H_34	Source of water in the rainy season?<br>☐ Treated pipe-borne water ☐ Mechanical well ☐ Ordinary well

	☐ Reservoir collecting rain water ☐ River/Lake/Dam ☐ Others
H_35	If other, please specify:
H_36	Source of water in the rainy season?<br>☐ Treated pipe-borne water ☐ Mechanical well ☐<br>Ordinary well<br>☐ Reservoir collecting rain water ☐ River/Lake/Dam ☐ Others
H_37	If other, please specify:

Household Assets. Does the Household have any of these amenities?

H_38	**Radio**	☐ Yes	☐ No	
H_39	If yes, quantity?	|___||___|		
H_40	**TV**	☐ Yes	☐ No	
H_41	If yes, quantity?	|___||___|		
H_42	**Video/CD/DVD player**	☐ Yes	☐ No	
H_43	If yes, quantity (of all)?	|___||___|		
H_44	**Landline phone**	☐ Yes	☐ No	
H_45	If yes, quantity?	|___||___|		
H_46	**Mobile phone**	☐ Yes	☐ No	
H_47	If yes, quantity?	|___||___|		
H_48	**Mobile phone**	☐ Yes	☐ No	
H_49	If yes, quantity?	|___||___|		
H_50	**Type of phones**	☐ Smartphone	☐ Regular cell phone	☐ Other
H_51	If other, please specify:			
H_52	**Purchase year of latest phone**	|___|___|/|___|___|/|___|___| (DD/MM/YY)		
H_53	**Fridge/Freezer**	☐ Yes	☐ No	
H_54	If yes, quantity?	|___||___|		
H_55	**Electric Stove**	☐ Yes	☐ No	
H_56	If yes, quantity?	|___||___|		

H_57	**Gas Stove**	☐ Yes	☐ No
H_58	If yes, quantity?	|___||___|	
H_59	**Solar plate**	☐ Yes	☐ No
H_60	If yes, quantity?	|___||___|	
H_61	If yes, what kind?		
H_62	**Solar lamp**	☐ Yes	☐ No
H_63	If yes, quantity?	|___||___|	
H_64	**Oil lamp**	☐ Yes	☐ No
H_65	If yes, quantity?	|___||___|	
H_66	**Gas lamp**	☐ Yes	☐ No
H_67	If yes, quantity?	|___||___|	
H_68	**Car**	☐ Yes	☐ No
H_69	If yes, quantity?	|___||___|	
H_70	**Motorbike**	☐ Yes	☐ No
H_71	If yes, quantity?	|___||___|	
H_72	**Tricycle**	☐ Yes	☐ No
H_73	If yes, quantity?	|___||___|	
H_74	**Bicycle**	☐ Yes	☐ No
H_75	If yes, quantity?	|___||___|	
H_76	**Desktop Computer**	☐ Yes	☐ No
H_77	If yes, quantity?	|___||___|	
H_78	**Laptop Computer**	☐ Yes	☐ No
H_79	If yes, quantity?	|___||___|	
H_80	**Bednets**	☐ Yes	☐ No
H_81	If yes, quantity?	|___||___|	

	STUDY CENTRE:	SeroCoV Study Forms<br>Case Report Form (Household)	I__II__I/I__II__I/I__II__I<br>(DD/MM/YY)

Protective measures

H_82	Did you take any measures to protect your household members?<br>☐ Yes ☐ No ☐ Not sure
H_83	What do you think should be done to protect household members?<br>☐ Use of masks for everyone<br>☐ Use of masks for individuals with symptoms<br>☐ Use of masks for older members or individuals with other diseases<br>☐ Not taking a meal together<br>☐ Not sharing personal objects<br>☐ Washing hands more regularly<br>☐ Avoiding contacts with other households' members<br>☐ Avoiding markets and other crowded places<br>☐ Cleaning household objects more regularly<br>☐ Other
H_84	If other, please specify:
H_85	Did you take any measures to protect the elderly/those with underlying conditions in your household?<br>☐ Yes ☐ No ☐ Not sure
H_86	If yes, how?<br>☐ Masks for vulnerable<br>☐ Masks for household members taking care of vulnerable<br>☐ Household members avoiding contact with vulnerable<br>☐ Children avoiding contacts with vulnerable<br>☐ Vulnerable eating alone<br>☐ Vulnerable not going out of the household<br>☐ Vulnerable washing hands more regularly<br>☐ Cutlery plate used only for vulnerable<br>☐ Other
H_87	If other, please specify:
H_88	In general, when did you take precaution measures?<br>☐ When SARS-CoV-2 / COVID-19 was first mentioned in the news<br>☐ After some cases happened in the neighbourhood<br>☐ After the first case in the household<br>☐ Since the beginning of the corona-pandemic<br>☐ Never<br>☐ Other
H_89	If other, please specify:

H. Household economic consequences:

H_90	How many household members could not work during the Corona epidemic?<br>I___II___I
H_91	Did it reduce the household income? ☐ Yes ☐ No ☐ Not sure
H_92	Have you had to borrow money to other family members or person outside the household because of the pandemic? ☐ Yes ☐ No
	Did you lend money to other households? ☐ Yes ☐ No

Household Assets. Does the Household have any of these amenities?

Responsibilities

Data transfer successful ☐ Yes ☐ No

Signature (fieldworker):

Observa

O_1	Blood sample taken? ☐ Yes ☐ No
O_2	Household member sampling ☐ Single ☐ Complete
O_3	Date I__II__I/I__II__I/I__II__I I__II__I (DD/MM/YYYY)
O_4	Remarks

	STUDY CENTRE:	SeroCoV Study Forms<br>Case Report Form (Household)	|_||_|/|_||_|/|_||_| (DD/MM/YY)

**O_5**	Field worker initials |__||__||__|
**O_6**	What is the type of housing?<br>☐ Apartment/Flat ☐ Single family house ☐ Compound house ☐ Other
**O_7**	If other, please specify
**O_8**	Nature of walls<br>☐ Hard ☐ Semi hard ☐ Banco improved ☐ Banco ☐ Straw ☐ Other
**O_9**	If other, please specify
**O_10**	Nature of floor<br>☐ Tiles ☐ Cement ☐ Beaten ☐ Ground ☐ Sand ☐ Natural floor/earths/sand ☐ Rudimentary floor (wood planks/plam, bamboo<br>☐ Finished floor (ceramic tiles, cement, carpet, etc.) ☐ Other
**O_11**	Nature of roof<br>☐ Concrete ☐ Sheet ☐ Tiles ☐ Beaten Ground ☐ Straw ☐ Others
**O_12**	If other, please specify
**O_13**	Horse Cart<br>Country-specific question (only Burkina Faso)<br>☐ Yes ☐ No
**O_14**	Data transfer successful ☐ Yes ☐ No

	STUDY CENTRE:	SeroCoV Study Forms<br>Case Report Form (Member)	|_||_|/|_||_|/|_||_| (DD/MM/YY)

m_01	**Has the participant / legal representative signed the informed consent?**
m_02	**If not, why?**
m_03	**If other, please specify**
m_04	**Household ID:**
m_05	**Member ID:**
	**Recruitment Date:** |___|___|/|___|___|/|___|___| (DD/MM/YY)
	**Data collector initials:** |__||__||__|

Participant information

m_06	Sex: ☐ Male ☐ Female
m_07	Age (in completed years): |__||__| in years
m_08	What is your highest educational qualification? *(ask only if above 14 years of age)*<br>☐ None ☐ Middle school/JHS ☐ WASSCE/O/A level<br>☐ Diploma/certificate ☐ Bachelor's degree ☐ Master's degree<br>☐ PhD/Fellowship ☐ Other
m_09	If other, please specify:
m_10	What is your employment situation?<br>☐ Employed ☐ Self-employed ☐ Farming<br>☐ Student/Intern/Apprentice ☐ House keeper/homemaker<br>☐ Retired<br>☐ Government employee ☐ Unemployed (health reasons)<br>☐ Unemployed (other reasons) ☐ Not applicable (under 18) ☐<br>Other
m_11	If other, please specify:
m_12	What is your main source of income?
m_13	Occupational Sector<br>☐ Health care ☐ Business/trade/Retail ☐ Education ☐ Transportation<br>☐ Agriculture ☐ Building and Construction ☐ Other Civil/Public servants<br>☐ Other
m_14	If other, please specify:
m_15	Do you have another source of income? ☐ Yes ☐ No

m_16	If yes, please specify:
m_17	Where do you spend most of your working time?<br>☐ Inside ☐ Outside ☐ Both
m_18	Do you work and live in the same area? ☐ Yes ☐ No
m_19	How long does it take to go to work?<br>☐ A few minutes ☐ Up to one hour ☐ Up to two hours<br>☐ More than two hours ☐ A whole day ☐ Other
m_20	If Other, please specify:
m_21	How do you get to work every day?<br>☐ Walk ☐ Bus ☐ Car ☐ Train ☐ Combination ☐ Other
m_22	If Other, please specify:
m_23	How many people do you come in contact with on your way to work?<br>☐ Less than 5 ☐ 5 to less than 10 ☐ 10 to less than 50<br>☐ 50 or more
m_24	How many people do you usually come into contact with in a day?<br>☐ Less than 5 ☐ 5 – 9 people ☐ 10 or more people
m_25	Have you been travelling outside of your resident city in 2020?<br>☐ Yes ☐ No
m_26	If yes, how often do you travel in general?<br>☐ Regularly ☐ Occasionally ☐ Rarely
m_27	If yes, how often?<br>☐ Once ☐ Daily ☐ Weekly ☐ Monthly ☐ Not sure
m_28	Where did you travel to?<br>☐ Within country ☐ International ☐ Both
m_29	How did you travel?<br>☐ By airplane ☐ Public Transport (bus, taxis etc.) ☐ Private vehicle ☐<br>Combination
m_30	Have you travelled to countries that have a high risk of SARS-CoV-2 transmission?<br>☐ Yes ☐ No ☐ Not Sure
m_31	Do you know a confirmed case / Have you come in contact with a confirmed case?<br>☐ Yes ☐ No ☐ Don't know
m_32	Are you pregnant? (*Ask only if above 14 years)*

	☐ Yes ☐ No ☐ Don't know

Medical history and risk perception

m_33	Do you have an underlying condition?<br>☐ Yes ☐ No ☐ Don't know
m_34	If yes, which one(s)? (*Multiple options allowed)*<br>☐ Lung disease (i.e, TBC) ☐ Diabetes ☐ Cortisol intake ☐ Heart diseases<br>☐ Obesity ☐ Immunosuppressive medication ☐ Hypertension<br>☐ Other
m_35	Do you smoke?<br>☐ Yes ☐ No ☐ Occasionally
m_36	Do you perceive yourself at risk for severe COVID-19 disease?<br>☐ Yes ☐ No ☐ Don't know

History of the Disease and Test

m_37	**In the past 2 weeks, have you had any of the following symptoms?** *(multiple options possible)*		
m_38	Fever ≥38°C	☐ Yes	☐ No
m_39	Fatigue	☐ Yes	☐ No
m_40	Sore throat	☐ Yes	☐ No
m_41	Cough	☐ Yes	☐ No
m_42	Chest pain	☐ Yes	☐ No
m_43	Shortness of breath	☐ Yes	☐ No
m_44	Loss of taste or smell	☐ Yes	☐ No
m_45	Other respiratory symptoms	☐ Yes	☐ No
m_46	**If yes, please specify:**		
m_47	Abdominal pain	☐ Yes	☐ No
m_48	Headache	☐ Yes	☐ No
m_49	Diarrhoea	☐ Yes	☐ No
m_50	What about **throughout the year**, have you had any of the following symptoms? *(Multiple options possible)*		
m_51	Fever ≥38°C	☐ Yes	☐ No
m_52	Fatigue	☐ Yes	☐ No
m_53	Sore throat	☐ Yes	☐ No
m_54	Cough	☐ Yes	☐ No
m_55	Chest pain	☐ Yes	☐ No
m_56	Shortness of breath	☐ Yes	☐ No
m_57	Loss of taste or smell	☐ Yes	☐ No
m_58	Other respiratory symptoms	☐ Yes	☐ No
m_59	**If yes, please specify:**		
m_60	Abdominal pain	☐ Yes	☐ No
m_61	Headache	☐ Yes	☐ No
m_62	Diarrhoea	☐ Yes	☐ No
m_63	Which places did you seek care in the past 2 weeks after developing any of the symptoms checked above *(Select all that apply)*<br>☐ Traditional healer ☐ Traditional self-medication ☐ Modern self-medication ☐ Private pharmacy ☐ Health care facility ☐ Other		

m_64	If other, please specify:
m_65	Which places did you seek care throughout after developing any the symptoms checked in C01? (Select all that apply)<br>☐ Traditional healer ☐ Traditional self-medication ☐ Modern self-medication ☐ Private pharmacy ☐ Health care facility ☐ Other
m_66	If other, please specify:
m_67	Were you tested for the coronavirus (swab)?<br>☐ Yes ☐ No ☐ Don't know
m_68	If yes, where did you get tested?<br>☐ Health care facility ☐ Diagnostic centre/Laboratory ☐ Other
m_69	If other, please specify:
m_70	How long did you wait to get the result?<br>☐ One day ☐ Less than a week ☐ More than a week<br>☐ Less than a month ☐ More than a month ☐ Still waiting for results<br>☐ Other
m_71	If other, please specify:
m_72	Was the test positive? ☐ Yes ☐ No ☐ Don't know
m_73	If yes, do you know where you got the disease from?<br>☐ Social contact ☐ Work contact ☐ Household contact ☐ Other<br>☐ Don't Know
m_74	If yes, were you in isolation after the test results were given?<br>☐ Yes ☐ No
m_75	If yes, where were you in isolation after the test result was given?<br>☐ Home, isolated from others ☐ Isolation centre ☐ In hospital<br>☐ Other
m_76	Were you in hospital for treatment? ☐ Yes ☐ No
m_77	If yes, did you receive any medication? ☐ Yes ☐ No

m_78	If yes, which medication did you receive?
m_79	Did you receive oxygen at any point during your treatment?<br>☐ Yes ☐ No ☐ Don't know
m_80	Did you fear for your life? ☐ Yes ☐ No
m_81	Did you have any costs for treatment that was not covered by health insurance?<br>☐ Yes ☐ No
m_82	If yes, could you tell us how much you paid for?<br>Consultations |__||__||__||__|
	Hospitalisations |__||__||__||__|
	Tests/medical examinations |__||__||__||__|
	Treatments |__||__||__||__|
	Transportation |__||__||__||__|
	Food/Accomodation |__||__||__||__|

Perceptions and information *(ask only if above 14 years of age)*

m_83	Among 100 individuals infected with COVID-19, how many do you think are dying from the disease?<br>Answer *(number)*: |__||__||__|
m_84	Was your salary maintained during the COVID-19 pandemic?<br>☐ Yes ☐ No ☐ Partly ☐ Don't know
m_85	What is your source of information about Coronavirus? *(Select all that apply)*<br>☐ Social media (e.g., Facebook) ☐ Radio ☐ TV ☐ Ministry of Health<br>☐ Webpages (e.g., Google top hits) ☐ Newspaper ☐ Health workers<br>☐ Family and friends
m_86	Which source do/did you trust most?<br>☐ Social media (e.g., Facebook) ☐ Radio ☐ TV ☐ Ministry of Health<br>☐ Webpages (e.g., Google top hits) ☐ Newspaper ☐ Health workers<br>☐ Family and friends

STUDY CENTRE:	SeroCoV Study Forms<br>Case Report Form (Member)	|_||_|/|_||_|/|_||_| (DD/MM/YY)

m_87	How have your living conditions changed since the beginning of the corona crisis?
m_88	Since the COVID-19 pandemic, have you changed your eating habits?<br>☐ Yes, totally ☐ Yes, a little ☐ No, not much ☐ No, not at all ☐ Don't know
m_89	Since the COVID-19 pandemic, have you made food deviations?<br>☐ Yes, totally ☐ Yes, a little ☐ No, not much ☐ No, not at all ☐ Don't know
m_90	Since the COVID-19 pandemic, have you drunk more alcoholic beverages than usual?<br>☐ Yes, totally ☐ Yes, a little ☐ No, not much ☐ No, not at all ☐ Don't know
m_91	Since the COVID-19 pandemic, have you had good access to basic necessities *(e.g., food and medication)?*<br>☐ Yes, totally ☐ Yes, a little ☐ No, not much ☐ No, not at all ☐ Don't know
m_92	How much confidence do you have in the information provided by the National Institute for Public Health about the novel coronavirus?<br>☐ Very confident ☐ Confident ☐ A little confident ☐ Not confident at all<br>☐ Undecided ☐ Don't know
m_93	Do you know about the advised measures help to limit the spread of SARS-CoV-2?<br>☐ Yes ☐ No ☐ Not sure
m_94	How much confidence do you have in the measures taken by the government to limit the spread of the novel coronavirus?<br>☐ Very confident ☐ Confident ☐ A little confident ☐ Not confident at all<br>☐ Undecided ☐ Don't know
m_95	Do you think that the advised measures help to limit the spread of the novel coronavirus?<br>☐ Yes ☐ No ☐ Not sure ☐ Maybe
m_96	Do you find it difficult to comply with the advised measures?<br>☐ No, not at all ☐ Maybe, a little difficult ☐ Yes, very difficult ☐ Undecided<br>☐ Don't know
m_97	If difficult, did/ do you find it difficult to: *(multiple options possible)*<br>☐ Maintain social distancing ☐ Wear face masks ☐ Not work<br>☐ Socially isolate ☐ Quarantine ☐ Frequently wash hands<br>☐ Other
m_98	Which measure was the most difficult to comply with?<br>☐ Maintain social distancing ☐ Wear face masks ☐ Not work<br>☐ Socially isolate ☐ Quarantine ☐ Frequently wash hands<br>☐ Other
m_99	Do people in your immediate environment comply with the advised measures?<br>☐ Yes ☐ No ☐ Don't know
m_100	Do you think that people should comply with the advised measures?<br>☐ Yes ☐ No ☐ Don't know

Could you please answer the following questions?

m_102	Over the last two weeks I have felt cheerful and in good spirits<br>☐ All of the time ☐ Most of the time ☐ More than half of the time<br>☐ Some of the time ☐ At no time
m_103	Over the last two weeks I have felt calm and relaxed<br>☐ All of the time ☐ Most of the time ☐ More than half of the time<br>☐ Some of the time ☐ At no time
m_104	Over the last two weeks I have felt active and vigorous<br>☐ All of the time ☐ Most of the time ☐ More than half of the time<br>☐ Some of the time ☐ At no time
m_105	Over the last two weeks I woke up feeling fresh and rested<br>☐ All of the time ☐ Most of the time ☐ More than half of the time<br>☐ Some of the time ☐ At no time
m_106	Over the last two weeks my daily life has been filled with things that interest me<br>☐ All of the time ☐ Most of the time ☐ More than half of the time<br>☐ Some of the time ☐ At no time
m_107	Were you worried about the health of family/ friends during the time of the SARS-Cov-2 outbreak?<br>☐ Not worried at all ☐ A little worried ☐ Undecided ☐ Worried ☐

For Office Use Only

Field Officer's initials: |__||__|
